# Ruminal In Vitro Protein Degradation and Apparent Digestibility of Energy and Nutrients in Sheep Fed Native or Ensiled + Toasted Pea (*Pisum sativum*) Grains

**DOI:** 10.3390/ani9070401

**Published:** 2019-07-01

**Authors:** Martin Bachmann, Christian Kuhnitzsch, Paul Okon, Siriwan D. Martens, Jörg M. Greef, Olaf Steinhöfel, Annette Zeyner

**Affiliations:** 1 Institute of Agricultural and Nutritional Sciences, Martin Luther University Halle-Wittenberg, Theodor-Lieser-Straße 11, 06120 Halle (Saale), Germany; 2 Saxon State Office for Environment, Agriculture and Geology, Am Park 3, 04886 Köllitsch, Germany; 3 Institute for Crop and Soil Science, Federal Research Centre for Cultivated Plants, Julius Kühn Institute (JKI), Bundesallee 58, 38116 Braunschweig, Germany

**Keywords:** field peas, ensiling, hydro-thermic treatment, nutrient digestibility, rumen-undegraded protein, *Streptomyces griseus* protease test

## Abstract

**Simple Summary:**

Pea grains may partially replace soybean or rapeseed meals and cereals in ruminant diets, but this is limited by high solubility of pea protein in the rumen. Hydro-thermic treatments such as toasting may stabilize the protein and shift digestion from the rumen to the small intestine. The effect of toasting of ensiled pea grains on rumen-undegraded protein was tested in vitro and on apparent digestibility of organic matter, gross energy, and proximate nutrients in a digestion trial with sheep. Ensiling plus toasting increased rumen-undegraded protein from 20 to 62% of crude protein, but it also increased acid detergent insoluble protein, which is unavailable for digestive enzymes in the small intestine from 0.5 to 2.6% of crude protein. Ensiling plus toasting did not, however, affect total tract apparent digestibility of organic matter, energy, crude protein, or any other nutrient fraction, nor did it alter the concentration of metabolizable energy or net energy lactation in the peas. The technique can be implemented on farms and might have a positive impact on field pea production.

**Abstract:**

Pea grains may partially replace soybean or rapeseed meals and cereals in ruminant diets, but substitution by unprocessed peas is limited by high ruminal protein solubility. The effect of combined ensiling and toasting of peas using a mobile toaster (100 kg/h throughput rate, 180 to 190 °C supplied air temperature) on rumen-undegraded protein (RUP) was tested in vitro using the *Streptomyces griseus* protease test. The effects of ensiling plus toasting on apparent digestibility of organic matter (OM), gross energy (GE), and proximate nutrients were examined in a digestion trial. Concentrations of metabolizable energy (ME) and net energy lactation (NEL) were calculated. Native peas had 38 g RUP/kg dry matter (DM), which was 20% of crude protein (CP). Rumen-undegraded protein increased three-fold after ensiling plus toasting (*p* < 0.001). Acid detergent insoluble protein increased five-fold. Apparent digestibility was 0.94 (OM), 0.90 (CP), and above 0.99 (nitrogen-free extract, starch, and sugars) and was not altered by the treatment. The ME (13.9 MJ/kg DM) or the NEL (8.9 MJ/kg DM) concentration was similar in native and ensiled plus toasted peas. This technique can easily be applied on farms and may increase RUP. However, it needs to be clarified under which conditions pea protein will be damaged.

## 1. Introduction

The production of non-genetically modified foods and animal feeds is increasingly prominent in the public and the political spotlights. In the livestock sector, soybean meal is, apart from rapeseed meal (after oil extraction), undoubtedly the most intensively used protein feed, but it nearly completely originates from genetically modified sources [1]. The European Union (EU-28) currently imports about 95% of the utilized soybeans [2]. National programs now intend to reduce soybean imports and partly replace the classical protein feeds by more cost-effective and sustainable indigenous protein plants such as lupines, faba beans, or field peas. This supports local feed production and nutrient cycles as well. Field pea grains combine high percentages of protein and starch—210 to 261 and 404 to 496 g/kg dry matter (DM), respectively [3,4,5]. Although post-extraction soybean and rapeseed meals have higher protein contents (508 to 564 and 365 to 411 g/kg DM, respectively [6,7]), they do not provide comparable amounts of starch or other non-structural carbohydrates. The one-to-one substitution of soybean meal and cereals by field pea grains was successful in dairy cows on moderate and high production levels and did not affect feed intake and (fat-corrected) milk yields as long as equal levels of rumen-undegraded protein (RUP) were considered [8,9]. However, continuously high and increasing levels of milk production require a better utilization of feed energy and nutrients. Starch, but especially protein, from field peas is highly degradable in the rumen when the grains are unprocessed, which is limiting small intestinal availability [10]. Increasing RUP might therefore enable the substitution of soybean or rapeseed meals in high-yielding animals [8].

Previous in vitro [11] and in vivo [12] studies demonstrated that the combination of ensiling and subsequent toasting of pea grains is suitable to obtain harvesting and storage stability regardless of the weather conditions. Contrary to our expectations, ensiling of pea grains still led to reduction of protein solubility and an increase of RUP in vitro [11]. Toasting of dry and unprocessed peas after harvesting did not decrease protein solubility, and did not increase RUP or post-ruminal crude protein (CP). Therefore, we further used the combination of ensiling and toasting. We hypothesized that hydro-thermic treatment (toasting) of ensiled field pea grains with 180 to 190 °C supplied air temperature (i.e., 85 to 90 °C grain temperature) and 100 kg/h throughput rate in the toaster would increase RUP without damaging the protein. Apparent digestibility of organic matter (OM), crude nutrient fractions, starch, sugars, and gross energy (GE) was expected to remain unaffected, which would (regarding pea protein) increase the amount that is available for digestion in the small intestine. The effect of ensiling plus toasting on RUP contents of field pea grains was tested in vitro using the *Streptomyces griseus* protease test. Treatment effects on apparent digestibility of energy, OM, and proximate nutrients were proven in a standard digestion trial with sheep. Additionally, the data were used to calculate GE, metabolizable energy (ME), and net energy lactation (NEL) of the pea treatments, applying official equations of the Society of Nutrition Physiology (GfE) [13,14] and the National Research Council (NRC) [15].

## 2. Materials and Methods

### 2.1. Pea Treatments

The field pea cultivar Alvesta (KWS SAAT SE, Einbeck, Germany) was used. It was grown and harvested in 2017 in Köllitsch, Saxony. A total quantity of 27.3 t DM of the native grains (i.e., as harvested; not ensiled or toasted) were re-moistened from 779 to 749 g/kg by adding 120 g of a homo-fermentative lactic acid bacteria inoculant including *Lactobacillus plantarum* and *Pediococcus acidilactici* strains (LAB; together 6.8 × 10^6^ colony forming units per g fresh matter; Josilac^®^ classic, Josera GmbH & Co. KG, Kleinheubach, Germany) in 200 L water (i.e., 0.6 g LAB/L), crushed using a Murska 2000 S2x2 (Murska, Ylivieska, Finland), and ensiled in a silage plastic bag (BAG Budissa Agroservice GmbH, Malschwitz, Germany) for 9 months. The grain silage had 749 g DM/kg, 34 g crude ash (CA)/kg DM, 189 g CP/kg DM, 20 g acid ether extract (AEE)/kg DM, and 63 g crude fiber (CF)/kg DM. Further ensiling characteristics are specified in Table 1. The aerobic stability of the field pea grain silage was tested following the procedure of H. Honig [16]. Subsequently, the ensiled grains were toasted using a mobile toaster (EcoToast 100, Agrel GmbH, Arnstorf, Germany) at atmospheric pressure with a throughput rate of 100 kg/h, 180 to 190 °C supplied air temperature, and 85 to 90 °C grain temperature.

### 2.2. In Vitro Estimation of RUP

The concentrations of RUP in native (i.e., not ensiled or toasted) and ensiled plus toasted pea grains were estimated in vitro using the *Streptomyces griseus* protease test [17] with 10 analytical replicates each. Briefly, 0.5 g of material was weighed into the 136 mL glass bottles, 40 mL of borate phosphate buffer consisting of 12.20 g/L NaH_2_PO_4_ × H_2_O + 8.91 g/L Na_2_B_4_O_7_ × 10H_2_O (pH 6.7 to 6.8) was added, and the solution was incubated for 1 h at 39 °C in a shaking water bath (80 rpm). A solution of nonspecific type XIV *Streptomyces griseus* protease (Sigma-Aldrich Chemie GmbH, Munich, Germany; ≥3.5 U/mg) with an activity of 0.58 U/mL was made following Licitra et al. [18]. The protease preparation combined aminopeptidase and caseinolytic activities [19]. One unit was defined to hydrolyze casein producing color equivalent (Folin-Ciocalteu reagent) to 1.0 μmol (i.e., 181 μg) of tyrosine per min at pH 7.5 and 37 °C. After 1 h, 3.63 and 3.55 mL of protease solution were added to the bottles (i.e., 24 U/g true protein) based on 179 and 175 g true protein/kg DM in native and ensiled plus toasted peas, respectively (calculated relative to a soybean standard with 493 g true protein/kg DM, which requires 10 mL of the solution) [17]. True protein was calculated from protein fractionation according to Licitra et al. [20] as CP—the non-protein nitrogen (A). The incubation time was set to 24 h considering a lag time of approximately 2 h [18]. After incubation, the bottles’ contents were filtered through Whatman #41 filter circles, and each was washed with 200 mL deionized water. The filters were air-dried, and nitrogen was determined in the residues and the blank filters using a FOSS Kjeltec^TM^ 8400 (FOSS GmbH, Hamburg, Germany).

The RUP content of the pea treatments was calculated as follows considering a weighed portion of 0.5 g of each treatment:

(1) $$\mathrm{RUP},\;g/\mathrm{kg}\;\mathrm{DM}=\left(\frac{\left(\left(N_{\mathrm{residue}}-N_{\mathrm{blank}}\right)\times 6.25\times 10\right)}{0.5\times {\mathrm{DM}}_{\mathrm{feed}}}\right)\times 10$$

where N_residue_ is nitrogen measured in the filter residues (mg); N_blank_ is mean nitrogen measured in the blank filters (mg); and DM_feed_ is the DM content of the native or ensiled plus toasted field pea grains (%).

### 2.3. In Vivo Determination of OM, GE, and Nutrient Digestibilities

The sheep used in this study were kept and cared for by the Research Centre for Agricultural and Nutritional Sciences, Martin Luther University Halle-Wittenberg, Wettin/Löbejün, Saxony-Anhalt, Germany. The experiment was carried out with approval by the Saxony-Anhalt Federal Administration Authority (approval no. 2-1524 MLU).

Eight adult Pomeranian coarsewool wethers were used as model animals for the digestion trial. All animals were clinically healthy and under regular veterinary supervision.

The composition of the offered diets is given in Table 2. Tap water was offered ad libitum. The feeding level of the sheep was close to the energy maintenance level [13,21]. The recommended feed protein content for digestibility trials with sheep is a minimum of 120 g CP/kg diet DM [22]. This was met during the current experiment. The diets were offered in two equal meals per day. The chemical compositions of the diet components and the mixed diets are given in Table 3.

The digestion trial was run as a difference test in two consecutive periods following the guidelines of GfE [22]. Each period consisted of 14 days adaptation to the diet followed by 6 days of total collection of feces. In total, 4 sheep received the native pea grains (i.e., not ensiled or toasted; 4 analytical replicates), and 5 sheep received the ensiled plus toasted peas (i.e., 5 analytical replicates). During the experiment, the sheep were individually housed in metabolic cages. All feed components were weighed for each mealtime and animal before the experiment started, and composited samples were taken. During total collection, the animals were fitted with harnesses for feces collection, which were emptied each morning prior the first meal. Daily defecation was quantified, and an aliquot of 0.2 was taken. Feed residuals were recorded. The fecal samples were composited individually per sheep per collection period. The feed and the fecal samples were stored dry or frozen at −20 °C, respectively.

The animals’ body weights were recorded in each period before adaptation, before feces collection, and at the end of the experiment. The initial body weight was 78 ± 9.7 kg. After a light decline, it was kept constant during the experiment (76 ± 7.5 kg before the first collection, 74 ± 7.8 kg before the second adaptation, 73 ± 11 kg before the second collection, and 73 ± 10 kg at the end of the experiment).

### 2.4. Analyses

Dry matter, CA, CP, AEE, CF, and Van Soest detergent fiber contents of feeds and feces were analyzed according to the German key book for feed analysis (VDLUFA methods no. 3.1, 4.1.1, 5.1.1 B, 6.1.1, 6.5.1, 6.5.2, 6.5.3, and 8.1) [23]. Neutral detergent fiber (NDF) was determined after 1 h treatment with heat stable amylase. Neutral detergent fiber and acid detergent fiber (ADF) were expressed exclusive of residual ash. Organic matter was calculated as OM = 1000 − CA, and the nitrogen-free extract (NFE) was calculated as NFE = OM − CP − AEE − CF. The amount of cellulose was calculated as cellulose = ADF − acid detergent lignin, and hemicellulose (HEM) was calculated as HEM = NDF − ADF. Neutral detergent insoluble CP (NDICP) was determined in the feeds according to VDLUFA method no. 4.13.1 [24]. Gross energy was determined in the feeds and the feces by bomb calorimetry using a C7000 Oxygen Bomb Calorimeter (IKA^®^ Werke, Staufen, Germany). Starch was determined in the feeds and the feces according to the amyloglucosidase method (VDLUFA method no. 7.2.5) [23]. The sugar content of the feeds and the feces was analyzed using anthrone reagent [25]. Enzyme-soluble organic matter (ESOM) was analyzed according to VDLUFA method no. 6.6.1 [23]. The protein fractions A (i.e., non-protein nitrogen), B1 (i.e., true protein, which is soluble in borate phosphate buffer at pH 6.7 to 6.8 but precipitable), B2 (i.e., true protein, which is insoluble in the borate phosphate buffer minus true protein, which is insoluble in neutral detergent), B3 (i.e., true protein, which is insoluble in neutral detergent but soluble in acid detergent), and C (i.e., true protein, which is insoluble in acid detergent) were determined in native and ensiled plus toasted field peas according to Licitra et al. [21] (method no. LKS FMUAA 1402015-11). For each fraction, residual nitrogen was determined according to the Kjeldahl method. The protein fractions were used to calculate the true protein content (i.e., B1 + B2 + B3 + C) and the soluble protein (i.e., A + B1). The protein insoluble in pepsin (CP_ip_) was also analyzed using the Kjeldahl method after 48 h of incubation in a pepsin-hydrochloric acid solution (method no. LKS FMUAA 1112014-07) [26]. The organic acids and the alcohols produced during the fermentation of the silage were determined by high performance liquid chromatography and refractive index detection (method no. LKS FMUAA 1662018-05) using a Shimadzu LC-20A Prominence (Shimadzu Corp., Kyoto, Japan) and a Hi-Plex H 8 µm column (300 × 7.7 mm; Agilent Technologies Inc., Santa Clara, CA, USA). All LKS methods were accredited according to DIN EN ISO/IEC 17025:2005.

### 2.5. Calculations and Statistical Analysis

Apparent digestibility coefficients of OM, CA, CP, AEE, CF, NDF, ADF, NFE, starch, sugars, and GE were calculated as the difference between intake and fecal output divided by the intake on a daily basis for each individual. Feed residuals were consistently lower than 2% of what was offered to the sheep. In accordance with the method prescription [22], feed residuals were thus not considered for digestibility calculation.

The current measured GE data and apparent digestibility coefficients were used to calculate GE, ME, and NEL according to GfE [13] as follows: GE, MJ/kg DM = 0.0239 × CP + 0.0398 × AEE + 0.0201 × CF + 0.0175 × NFE; ME, MJ/kg DM = 0.0312 × digestible AEE + 0.0136 × digestible CF + 0.0147 (digestible OM − digestible AEE − digestible CF) + 0.00234 × CP; and NEL, MJ/kg DM = 0.6 (1 + 0.004 (q − 57)) × ME, where q = ME/GE × 100. These equations were derived from the large data pool of total metabolic trials provided by the Institute for Animal Nutrition “Oskar Kellner” in Rostock (Germany) [27]. Moreover, GE, ME, and NEL were calculated on the basis of crude nutrient analyses using equations provided by GfE [13,14] and NRC [15].

Statistical analysis was performed using SAS 9.4 (SAS Institute Inc., Cary, NC, USA). The pooled *t*-test was used to compare RUP estimates and digestibility coefficients between the field pea treatments at a significance level of *p* < 0.05. Homogeneity of the sample variances was confirmed using the folded *F*-test, and the studentized residuals were confirmed to have Gaussian distribution using the UNIVARIATE procedure.

## 3. Results

**Figure 1:**
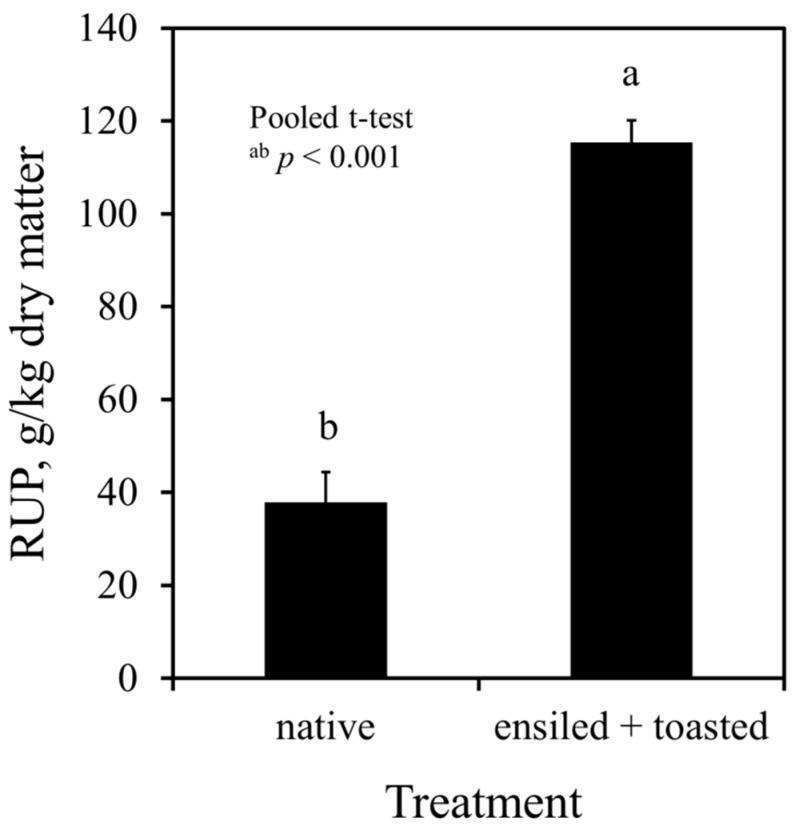
In vitro estimation of rumen-undegraded protein (RUP) in native and ensiled + toasted field pea grains using *Streptomyces griseus* protease (3.63 and 3.55 mL of 0.58 U/mL protease solution, respectively, and 24 h incubation time); native peas had 779 g DM/kg, 186 g CP/kg DM, and 179 g TP/kg DM; ensiled + toasted peas had 970 g DM/kg, 186 g CP/kg DM, and 175 g TP/kg DM; CP = crude protein, DM = dry matter, TP = true protein.

Native field pea grains had 38 g RUP/kg DM, which was 20% of CP. The concentration of RUP was increased to a three-fold in the ensiled plus toasted grains (115 g/kg DM, i.e., 62% of CP; *p* < 0.001), as shown in Figure 1. The protein fractionation procedure revealed that, after toasting the ensiled grains, protein solubility was five-fold decreased, soluble protein (B1) was nine-fold decreased, and the insoluble fractions B2 and B3 were two-fold and 27-fold increased, respectively (Table 4). The CP_ip_ was slightly increased. However, the C fraction was also increased to a five-fold after ensiling plus toasting (Table 4).

The apparent digestibility coefficients determined for OM, CA, CP, CF, ADF, NFE, starch, sugars, and GE did not differ between the treatments (Table 5). Native and ensiled plus toasted field peas did differ in AEE digestibility by tendency (0.49 vs. 0.61; *p* = 0.0548). They significantly differed in apparent digestibility of the NDF fraction (0.69 vs. 0.81; *p* < 0.05; Table 5).

Gross energy contents measured by bomb calorimetry and calculated according to GfE [13] were similar in the native and the ensiled plus toasted field peas (18.4 and 18.3 vs. 18.6 MJ/kg DM; Table 6). The native field peas had a digestible energy concentration of 16.7 MJ/kg DM, and the ensiled plus toasted peas had a digestible energy concentration of 16.6 MJ/kg DM, which was calculated based on measured GE and GE digestibility. The ME contents calculated on the basis of measured GE and nutrient digestibilities were 13.8 and 13.9 MJ/kg DM in native and ensiled plus toasted peas, respectively. A similar ME content was calculated in the native peas using GfE and NRC equations. In ensiled plus toasted peas, ME was slightly underestimated by 0.2 MJ/kg DM using GfE and by 0.4 MJ/kg DM using NRC equations (Table 6). This was similar with the NEL contents (Table 6).

## 4. Discussion

The social, the political, and the scientific interest in using regionally grown legumes as protein feeds for farm animals is increasing. Faba beans, field peas, lupines, clover, or lucerne can offer an alternative to soybean meal and rapeseed meal in Europe. Using field peas as at least a partial replacement of soybean or rapeseed meals and/or cereals was possible without addressed digestibility reduction, performance depression, or impaired quality of the end products in growing and finishing pigs [28], broiler chickens [29], laying hens [30], growing lambs and beef cattle [31,32], and dairy cows [8,9]. However, a complete replacement was not always successful in either monogastric [33,34] or ruminant livestock [8,32] when the peas remained unprocessed. A main factor that is limiting the substitution efficiency of pea grains is the higher ruminal protein solubility compared to soybean [35] or rapeseed meal. The RUP content of unprocessed peas is around 20% of CP, but in post-extraction soybean and rapeseed meals, 25 to 69% of CP was reported [36,37,38,39]. Moreover, ruminal starch degradation of unprocessed peas is approximately 56%, while it is 44% in maize and 83% in barley [40].

Ensiling may generally lead to proteolysis and carbohydrate degradation [41,42,43] but should not affect ruminal degradation of protein, starches, and other carbohydrates as long as no perishable fermentation (e.g., yeast fermentation) and concomitant heating occurs. Despite a good sensorial quality, the current pea grain silage was not reduced in pH (6.1), and ethanoic acid and ethanol contents were increased compared to previously used silages (by 1.4 and 6.8 g/kg DM) [11]. However, the proportion of true protein was comparable [11,12].

In native pea grains, storage proteins are mainly composed of the globulins 7S vicilin and 11S legumin, which are soluble to more than 70% in the digestive tract (74% in this study) [11,44]. Heat or heat plus pressure treatments such as dry roasting, steam flaking, autoclaving, toasting, extrusion, or expander treatment clearly decrease protein degradability in the rumen [4,5,6,10,45,46,47,48]. Feed protein structures are stabilized by complex Maillard reactions [46,47,49,50,51,52,53]. Next, proteolysis in the rumen can be inhibited or slowed down, but total tract digestibility of OM or protein should not be affected [4,54]. These effects largely depend on the type and the conditions of the treatment (e.g., temperature, throughput rate/duration of heat exposure, feed quantity, and moisture content) [11,53]. The native field pea grains in our study had a RUP content of about 20% of CP, which was similar to what Masoero et al. [5] measured. Then, RUP tripled in the ensiled plus toasted pea grains. Protein solubility decreased to one-fifth, B1 to one-ninth, and especially B3 increased by 27 times. We, however, also found that the C fraction increased five-fold after ensiling plus toasting, which might point to some protein damage.

The determination of total tract apparent digestibility of OM and crude nutrients is the official method to obtain ME and NEL of feedstuffs in Germany [13,21]. In native peas, total tract apparent digestibility coefficients of OM, GE, and proximate nutrients were similar to those reported by the German Agricultural Society (DLG) [39]. In our case, sheep were fed slightly below the maintenance requirement, which might have caused a minor overestimation of digestibility. Total tract apparent digestibility of OM, CA, CP, CF, ADF, NFE, starch, sugars, and GE was not affected by ensiling plus toasting, whereas AEE and NDF digestibility increased by approximately 10%. Total tract apparent digestibility does not allow conclusions on partial (e.g., ruminal) nutrient digestibility. The latter, however, might be altered by ensiling and toasting of peas, even if total tract digestibility remains unaffected. The increase in AEE and NDF digestibility was probably more an analytical issue and not a physiological response. Increased AEE digestibility was probably caused by the low AEE contents in both feed and feces, which needs to be set into relation to the inherent analytical error. Following ensiling plus toasting, NDICP was increased by approximately 30% of NDF. This probably led to an overestimation of the NDF intakes by the sheep and thus to an overestimation of NDF digestibility. When we reduced the NDF contents in the feed by the respective NDICP amounts, apparent digestibilities of NDF were 0.68 ± 0.063 and 0.74 ± 0.063 in native and ensiled plus toasted peas, respectively (*p* = 0.22). Although the samples were treated with amylase during NDF analysis, it is still possible that parts of starch contributed to an overestimation of the NDF intakes, at least when parts of the starch from the feed were altered by the feed treatment. Fiber, ash, and AEE digestibility coefficients had a large variation among the animals (standard deviation ranged from 0.05 to 0.20). Thus, the apparent increase of AEE and NDF digestibility was due to analytical uncertainties.

No effect of ensiling plus toasting on ME or NEL estimations was found. The available equations for ME and NEL estimation provided by the GfE [14] and the NRC [15] had a high conformity with calculations based on measured GE and ME calculated on the basis of measured GE and nutrient digestibilities.

## 5. Conclusions

The combination of ensiling and toasting of field pea grains on farms using a mobile toaster with 100 kg/hour throughput rate and 180 to 190 °C temperature of the supplied air (i.e., 85 to 90 °C grain temperature) led to a three-fold increase of RUP. However, boundary conditions for heat damage of proteins have to be clarified. Protein fractions that are fully insoluble to digestive enzymes (e.g., CPip or acid detergent insoluble protein) also increased after ensiling plus toasting, which should be avoided. Total tract apparent digestibility of OM, CA, CP, CF, ADF, NFE, starch, sugars, and GE was not affected by ensiling plus toasting. The apparent increase in AEE digestibility was probably an analytical issue. The NDF apparent digestibility probably increased due to increasing amounts of NDICP after toasting. Contents of GE, ME, and NEL remained unaffected. A high conformity was found among GE, ME, and NEL, which were measured, calculated, or estimated using GfE or NRC equations, respectively.

## Figures and Tables

**Table 1:** Fermentation characteristics of the field pea grain silage.

pH ^1^	6.1
pH ^2^	6.3
Lactic acid	2.3
Acetic acid	0.3
Propionic acid	<0.2
iso-Butyric acid	<0.3
n-Butyric acid	<0.1
iso-Valeric acid	<0.1
n-Valeric acid	<0.1
Ethanol	9.4
1,2-Propanediol	<0.3
1-Propanol	<0.4
Aerobic stability	≥7

The fatty acid and alcohol concentrations are given in g/kg dry matter, and the aerobic stability is given in days until the temperature difference between material and ambient exceeds 3 °C. ^1^ After ensiling. ^2^ After 7 days of aerobic storage.

**Table 2:** Composition of mixed diets offered during the experiment.

Component (g/Day as Fed)	Control Diet	Test Diet (Native Peas)	Test Diet (Ensiled + Toasted Peas)
Lucerne (chopped)	450	225	225
Barley (crushed, Ø 3.5 mm)	450	225	225
Wheat straw (chopped, Ø 6.0 mm)	100	50	50
Native peas	0	500	0
Ensiled + toasted peas	0	0	500
Mineral feed ^1^	10	10	10

^1^ basu-kraft^®^ Top-Mineral (BASU Heimtierspezialitäten GmbH, Bad Sulza, Germany).

**Table 3:** Chemical composition of the feeds and the diets used in the experiment.

	Lucerne	Wheat Straw	Barley	Native Peas	Ensiled + Toasted Peas	Mixed Diet (Control)	Mixed Diet (Including Native Peas) ^1^	Mixed Diet (Including Ensiled + Toasted Peas) ^1^
Dry matter	927	945	888	779	970	912	851	942
Crude ash	71	71	22	31	32	50	41	41
Organic matter	929	929	978	969	968	950	959	959
Crude protein	146	46	115	186	186	122	152	155
Acid ether extract	21	10	25	13	10	22	18	16
Starch	8	14	537	533	496	240	375	372
Sugars	47	12	42	77	38	41	58	40
Crude fiber	363	448	52	62	61	236	157	146
Neutral detergent fiber	524	843	210	128	197	420	285	305
Acid detergent fiber	389	506	64	80	79	259	176	166
Acid detergent lignin	85	59	8	5	15	49	29	31
Cellulose	56	447	304	75	64	210	147	135
Hemicellulose	135	337	146	48	118	161	109	139
Nitrogen-free extract	399	425	786	708	711	570	632	642
ESOM	555	344	880	953	944	675	803	814
Gross energy	19.1	17.9	18.6	18.4	18.3	18.8	18.6	18.5

ESOM = enzyme-soluble organic matter. ^1^ Pea treatments were included at an amount of 500 g dry matter per day. Dry matter (DM) is given in g/kg, gross energy is given in MJ/kg DM, and all other analytes are given in g/kg DM.

**Table 4:** Crude protein composition of the field pea treatments.

	Native Peas	Ensiled + Toasted Peas
Crude protein (CP)	186	186
True protein ^1^	179	175
Protein solubility ^2^	74	16
Protein fraction A	6.5	9.0
Protein fraction B1	67.7	7.2
Protein fraction B2	24.5	56.7
Protein fraction B3	0.9	24.5
Protein fraction C	0.5	2.6
CP_ip_	5.2	7.2
NDICP	1.1	61.6

A = non-protein nitrogen, B1 = buffer-soluble true protein, B2 = buffer-insoluble true protein—true protein that is insoluble in neutral detergent, B3 = true protein that is insoluble in neutral detergent, but soluble in acid detergent, C = true protein that is insoluble in acid detergent, CP_ip_ = protein insoluble in pepsin, NDICP = neutral detergent-insoluble CP. ^1^ True protein was calculated as CP − A. ^2^ Protein solubility was calculated as A + B1. CP, true protein, and NDICP are given in g/kg DM, and protein solubility, the protein fractions (A, B1, B2, B3, and C), and CP_ip_ are given in % of CP.

**Table 5:** Total tract apparent digestibility of energy and proximate nutrients in native and ensiled + toasted field pea grains.

	Native Peas	Ensiled + Toasted Peas
Organic matter	0.94 (0.019)	0.94 (0.026)
Crude ash	0.38 (0.200)	0.39 (0.140)
Crude protein	0.90 (0.033)	0.89 (0.042)
Acid ether extract	0.49 (0.049)	0.61 (0.088)
Crude fiber	0.61 (0.055)	0.65 (0.078)
Neutral detergent fiber	0.69 (0.059) ^b^	0.81 (0.055) ^a^
Acid detergent fiber	0.65 (0.069)	0.66 (0.074)
Nitrogen-free extract	0.99 (0.008)	0.99 (0.015)
Starch	1.00 (0.0007)	1.00 (0.001)
Sugars	1.00 (0.003)	0.99 (0.006)
Gross energy	0.91 (0.022)	0.91 (0.027)

Standard deviations are given in parentheses. ^ab^ Different superscripts mark significant differences between the treatments (*p* < 0.05).

**Table 6:** Gross energy (GE), metabolizable energy (ME), and net energy lactation (NEL) in native and ensiled + toasted field pea grains.

	Native Peas			Ensiled + Toasted Peas		
	Measured/Calculated ^1^	GfE ^2^	NRC ^3^	Measured/Calculated ^1^	GfE ^2^	NRC ^3^
GE	18.4	18.6	n.a.	18.3	18.6	n.a.
ME	13.8 ^4^	13.9	13.9	13.9 ^4^	13.7	13.5
NEL	8.9 ^4^	8.9	9.0	8.9 ^4^	8.8	8.7

n.a. = not applicable. ^1^ Calculated on the basis of measured GE concentrations and apparent digestibility of crude nutrients according to GfE [13]. ^2^ Calculated according to GfE [13,14]. ^3^ Calculated according to NRC [15]. ^4^ Given as the mean of 4 and 5 measurements in native and ensiled + toasted peas, respectively. All items are given in MJ/kg dry matter.
